# Transcatheter mitral and tricuspid therapy: outcomes in female patients

**DOI:** 10.1007/s12055-025-02069-5

**Published:** 2025-10-10

**Authors:** Gry Dahle

**Affiliations:** Department of Cardiothoracic Surgery, Oslo University Hospital, Sognsvannsveien 20, Oslo, 0372 Norway

**Keywords:** Mitral and tricuspid devices, Mitral and tricuspid trials, Sex distribution

## Abstract

Development of mitral and tricuspid devices has exploded, although the number of procedures is still limited. Women are under-represented in clinical trials, and in many trials, outcomes are not specified for sex. In mitral and tricuspid trials, the sex distribution is more even than in transcatheter aortic valve implantation (TAVI) trials. In these trials, in general, females are older, have more bleeding complications, and have less kidney injury than men. Procedural results are good. Long-term observation is still not available because of short observation time. In tricuspid procedures, other factors than device success play a role, like right heart failure, pulmonary hypertension, and atrial fibrillation. We should, for the future, focus more on differences in outcome between men and women to better customize treatment options.

**Graphical Abstract:**

Transcatheter mitral and tricuspid interventions, outcomes in women. Generated by AI.

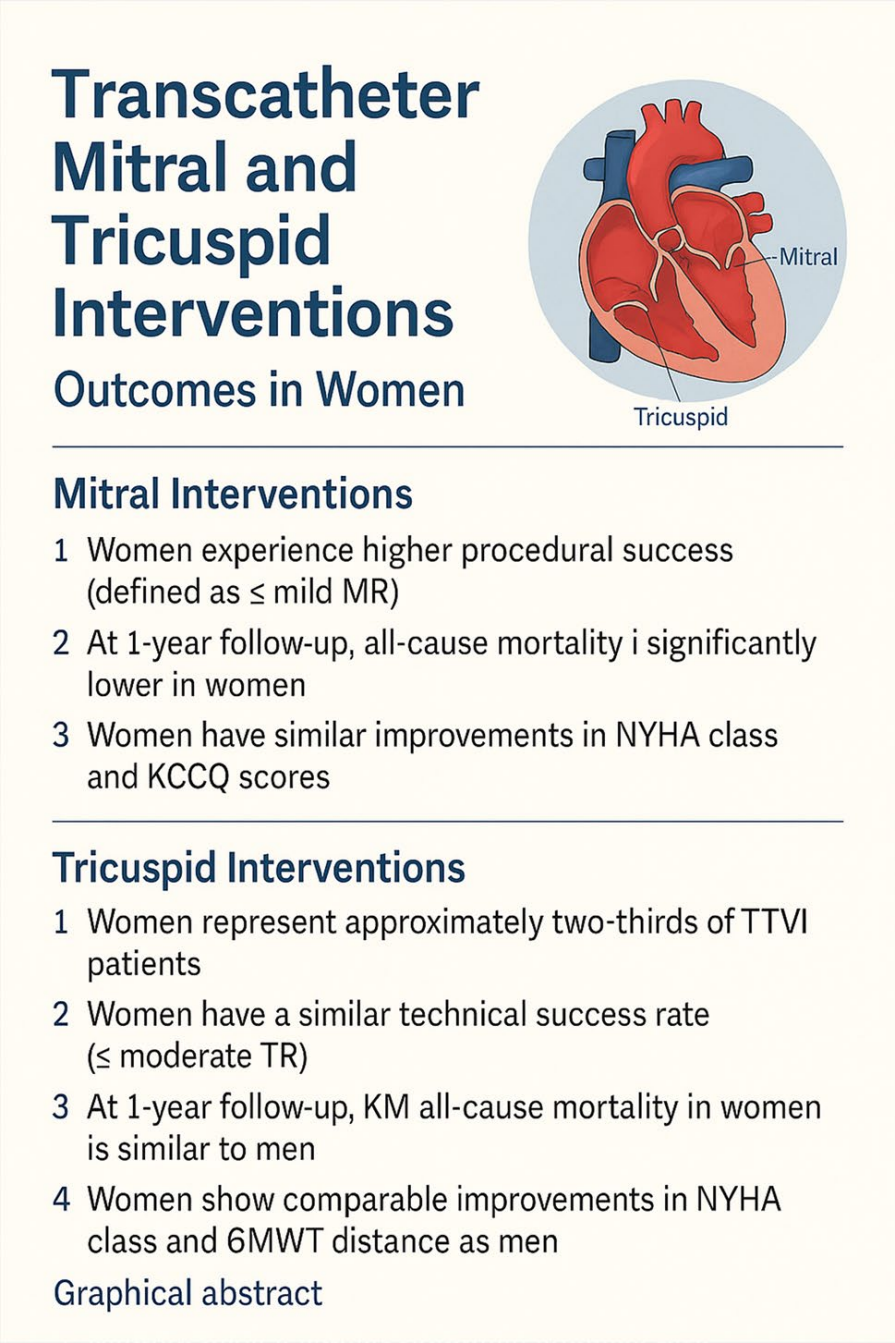

## Introduction

Transcatheter mitral and tricuspid therapies have emerged as feasible treatment options for patients deemed too high risk for conventional surgery. While transcatheter aortic valve implantation (TAVI) is now established as the standard of care in high-risk and intermediate-risk aortic stenosis, the mitral and tricuspid frontiers have proved to be more challenging. In this narrative review, the difference in outcome in men and women after transcatheter mitral valve (MV) and tricuspid interventions will be described.

First, MV repair with Mitraclip™ (Abbott, Abbott Park, IL, USA) was performed in 2003 on a female patient, Octalina Mendoza; the outcome was excellent [1]. The Chief Executive Officer (CEO) of Evalve™ company that is the precursor of Mitraclip™ was also female: Carolyn Powel. Then, the Endovascular Valve Edge-to-Edge Repair Study (EVEREST) trials comparing Mitraclip™ and surgery were performed. Here, female inclusions were 41% and 38% [2–4]; percutaneous repair was less effective than surgery, but the procedure was associated with similar clinical outcomes and superior safety.

Development of mitral and tricuspid devices has exploded, although the number of procedures is still limited.

## Under-representation of women in cardiovascular clinical trials

In cardiovascular (CV) research, women remain under-represented as participants and investigators [5–7]. In cardiovascular device studies, women have been under-represented more generally, and this does not reflect the underlying sex distribution in the disease. Burgess et al. found that the participation to prevalence ratio (PPR) for women in CV trials (the Placement of Aortic Transcatheter Valves (PARTNER) trials [8], Corevalve, Surtavi and Evolut low risk trials [9] was 41% [6]. In landmark clinical trials, 5% of all listed authors were women [5]. When evaluating landmark transcatheter mitral valve repair (TMVr) trials, 13% of all senior or first authors were women, and 15% of all listed authors were women [5]. When women are underpowered to detect important sex-based interactions, clinical trials cannot have an impact on the generalizability of trial data and sex-specific scientific evidence. Addressing the under-representation of women in clinical trials is an important step toward closing the data gap.

## Sex differences in mitral valve surgery and interventions

TMVr has been evaluated for sex-related differences in outcomes, revealing several key findings:

- Mortality rates: Studies indicate no significant 30-day and 1-year mortality rates between female and male patients undergoing MitraClip™ [10].
- Procedural success and complications: Both sexes achieve high procedural success rates with comparable reductions in mitral regurgitation severity. However, men may experience higher rates of acute kidney injury postoperatively, while women might have a slightly increased incidence of major bleeding [11].
- Functional outcomes: Post TMVr, women and men show significant improvements in functional status and quality of life. Nonetheless, women tend to have shorter 6-min walk distance both before and after the procedure, suggesting differences in exercise capacity [12].

MV disease is sex specific. This underlines the value of sex-specific evaluation in patients presenting with MV disease. Sex-specific differences in mitral pathology and perioperative factors contribute to a disparity in MV repair outcomes [13]. Women are more likely to have complex mitral pathology (e.g., anterior or bi-leaflet prolapse, mixed mitral lesions, or mitral annular calcification (MAC) [14].

The absence of indexed echocardiographic mitral regurgitation severity parameters contributes to delayed intervention, and females are often more symptomatic and present further along the disease course, resulting in higher-risk operations and interventions. Diagnostic criteria and sex-specific diagnostic criteria require further studies [14]. Females are likely to receive disproportionately less MV repair and experience more heart failure (HF) and worse early postoperative outcomes compared to their counterparts [15]. This reflects their more complex morphology. The pathophysiology of MV disease affects the outcomes of surgical and catheter repair. Females have more leaflet thickening/dysplasia and more complex mitral regurgitation (MR) pathophysiology involving anterior or leaflet prolapse, while males present more commonly with isolated posterior leaflet prolapse and are more suitable for transcatheter edge-to-edge repair (TEER). Infective endocarditis (IE) generally affects more men than women, except for affecting the MV. The higher rate of MV IE in women may be due to underlying native valve disease. MAC, which is associated with mitral stenosis (MS) and mixed MS/MR, is more prevalent in women, despite women generally having fewer risk factors for atherosclerosis, which have been linked to MAC progression [16]. MV intervention in the setting of MAC is surgically challenging; TEER is impossible, though transcatheter mitral valve implantation (TMVI) may be a solution. However, in these cases, there may be an issue with neo-left ventricular outflow tract (neoLVOT) [17].

In secondary MR, ischemic MR, associated with coronary artery disease disproportionately affects men [18], and in these patients, there is a lack of survival benefit addressing the MV in addition to percutaneous coronary intervention/coronary artery bypass grafting (PCI/CABG) (Table 1).

**Table 1:** Summarized outcomes for females in mitral valve interventions

Outcome category	Findings in women	Notes
Procedural success	Comparable to men, slightly lower in anatomically challenging cases	Anatomical differences (smaller annulus, leaflet length) may impact outcomes
30-day mortality	Similar of slightly lower than men	Studies show non-inferior results despite higher baseline comorbidities
1-year mortality	Similar to men	No significant sex-based difference in survival
Major bleeding	Higher incidence vs. men	Possibly due to body size and vascular access
Vascular complications	More frequent due to smaller vessel size	More common in women due to anatomical factors
Acute kidney injury (AKI)	Lower incidence vs. men	Men have higher baseline risk
Improvement in NYHA class	Significant improvement, similar to men	Post-TMVR functional status typically improves in both sexes
6-min walking distance	Improved but often lower than men post-procedure	Women may begin with shorter baseline distance
Quality of life (KCCQ score)	Significant improvement, comparable to men	KCCQ improvements are robust and clinically meaningful

At the time of MV surgery/intervention, women have a greater burden of comorbidities, reflected in older age and a higher prevalence of hypertension, respiratory failure, renal insufficiency, anemia, history of stroke/transient ischemic attack, HF, and urgent admission [19]. Procedural risk in women is further exacerbated by increased requirements for additional procedures to address sequelae of advanced MV disease, with higher rates of atrial fibrillation.

Percutaneous Repair with the MitrClip Device fro Severe Functional/Secondary Mitral Regurgitation in France (MITRA-FR) and COAPT are two landmark randomized trials of TEER outcomes of functional MR. The trials had 78.9% and 66.6% males in the device arm, respectively [20, 21]. In the larger COAPT study, the effectiveness endpoint of HF hospitalization within 24 months favored TEER among the male subgroup (HR 0.44, 95% CI 0.32–0.61), but did not reach significance in the female subgroup.

A meta-analysis demonstrates that men undergoing TEER have worse preoperative diseases (diabetes mellitus, coronary artery disease, renal failure, and myocardial infarction) while they have superior postoperative New York Heart Association (NYHA) class at 1 year. There were no significant differences in other indexes between men and women [22]. A single-center cohort study showed that although women experienced more recurrence of severe MR post-TEER, this was not associated with any significant difference in survival at 2 years [23]; in fact, there is a suggestion that TEER may disproportionately benefit women. Moreover, TEER in females is performed with lower costs per quality-adjusted life year gained than their male counterparts, likely due to longer life expectancy in women [24]. There is also a trend that heart valve interventions with males tend to have improved outcomes after surgical intervention, and females experience equivalent or improved outcomes after transcatheter interventions (Table 2).

**Table 2:**
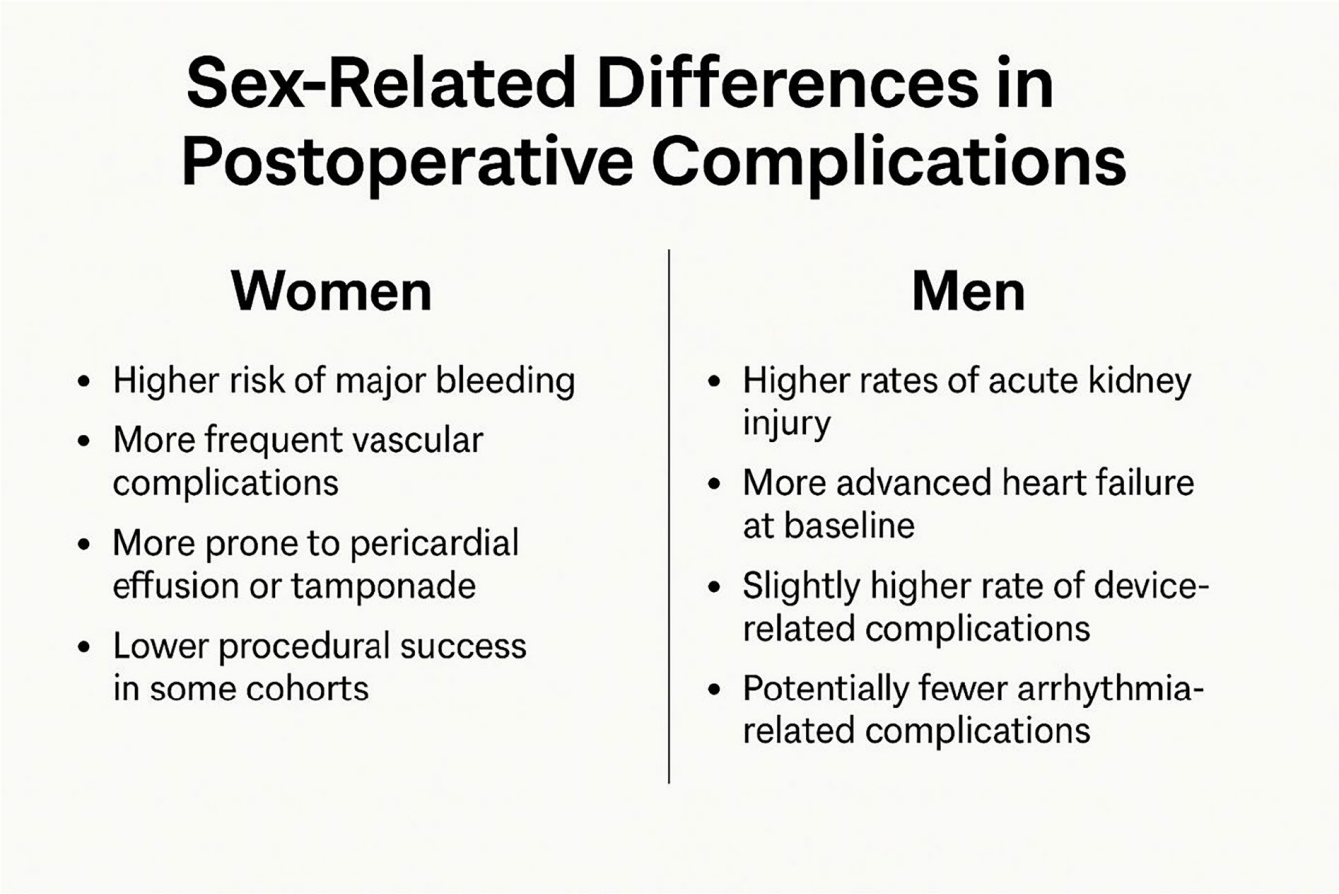
Sex-related differences in post mitral interventional complications

In most mitral device studies, females are under-represented, like in TAVI trials. Especially in the MV implantation, most studies have around 60% male; this is for the TENDER trial and for the CE mark trial for Tendyne™ (Abbott, Minneapolis, USA) [17, 25]. The results in the TMVI trials are not divided according to sex. The reason for more males may be anatomy, with too small neoLVOT in females and also small anatomy in general.

## Sex differences in tricuspid interventions

There is a growing interest in the tricuspid valve and for transcatheter tricuspid interventions, and these have improved treatment options.

Tricuspid valve disease constitutes a major valvular heart condition that is receiving highlighted attention due to tailored treatment options and sex-specific differences in the treatment outcome. The burden of tricuspid regurgitation (TR) is high in an aging population. Women live longer than men. This may be the reason for higher mortality and morbidity, in addition to more progressive disease.

Functional TR (due to annular dilatation and right heart remodeling) is more common than primary (organic) TR. The interventions may be repair or replacement. Tricuspid surgery is associated with high risk, and we do not have the Society of Thoracic Surgeons (STS) risk calculator to help calculate the risk of the procedure.

For primary regurgitation, the distribution between sexes seems almost equal, and even large studies on imaging differences between sexes are not described [26, 27].

If we use the STS risk calculator for TVR, the mortality risk for a 76-year-old male with no other risks is 1.89% and morbidity 11.3% versus 2.4% and mortality 12.8% for a female at the same age. For repair, the numbers are 1.32% and 8.68% for male and 1.69% mortality and 9.83% morbidity for females.

While both sexes benefit significantly from tricuspid interventions, women may face more bleeding and vascular risks, while men often have worse baseline cardiac function and are more prone to acute kidney injury and arrhythmias.

In tricuspid intervention trials, age is higher than in mitral and TAVI trials, and the proportion of women is higher. The prognosis of untreated severe TR is poor, and TR is associated with worsening HF and independently predicts mortality.

Tricuspid valve interventions are often underused; the most commonly used transcatheter tricuspid intervention for right heart failure (RHF) despite optimal medical therapy (OMT) is tricuspid transcatheter edge-to-edge repair (T-TEER), starting in 2016 [28]. However, many patients are rendered unsuitable for T-TEER as they present late with large coaptation gaps, leaflet tethering, and torrential TR. Transcatheter valve replacement (TTVR) has emerged as an attractive alternative. However, real-world clinical experience with patient screening for TTVI is associated with a screening failure of 60–70%, limiting the intervention [29] (please see Tables 3, and 4 for an overview).

**Table 3:** Implications for findings for transcatheter tricuspid treatment in women

Category	Findings in women	Implications
Baseline characteristics	Older age, more isolated TR, higher prevalence of right heart failure symptoms	Need for earlier referral and sex-specific consideration
Procedural success	High procedural success: anatomical factors may favor leaflet grasping	Good candidates for TTVI; procedure is technically feasible in most women
Short-term outcomes	Comparable slightly lower complication rates than men	Safety of TTVI supported; similar in-hospital and 30-day outcomes as men
Mid-/long-term outcomes	Improved survival and NYHA class, especially with isolated TR and preserved RV function	Potential better long-term benefit; supports broader use in symptomatic women
Imaging and anatomy	Smaller annuli, better leaflet coaptation; but large coaptation gaps can be challenging	Imaging quality of ten good, but personalized planning needed
Risk stratification	Traditional models underestimate morality risk in women	Development of sex-specific risk models needed
QOL	Greater symptom relief and functional improvement post-procedure	Supports inclusion of QOL metrics in assisting TTVI success in women

**Table 4:**
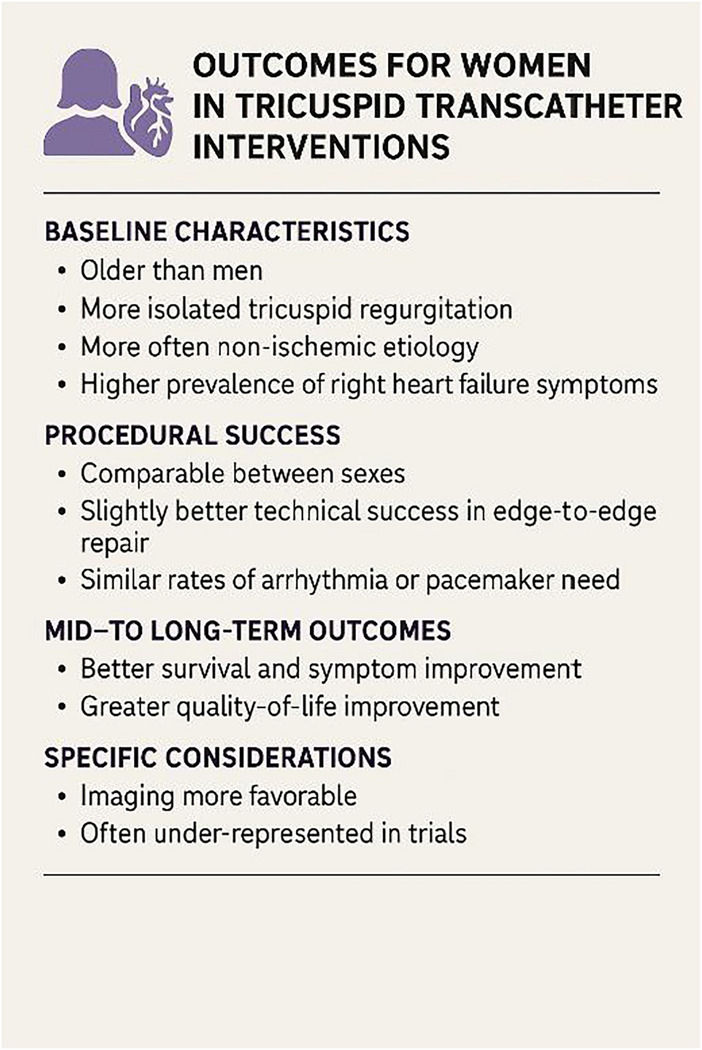
Outcomes for women in tricuspid interventions, generated by AI

### Procedural success and survival rate

In a JACC study of 702 patients with severe TR, treated with TTVI, there were no significant differences between women and men in terms of procedural success and 2-year survival rate, 69.9% vs. 63.7%, despite significant differences in cardiac volumetrics and comorbidities [30]. The men were more often diagnosed with coronary artery disease, and the underlying etiology for TR was predominantly secondary ventricular issues (64.6% for men vs. 50% in women), whereas women more often presented with atrial etiology (41.7% vs. 24.4% in men).

### Heart failure

RHF in women has some unique considerations compared to men due to anatomical, hormonal, and comorbid differences. Women typically have smaller right ventricles and lower baseline pulmonary artery pressures, so that any rise in afterload (due to pulmonary hypertension (PAH)) may lead to faster decompensation. Estrogen is thought to have protective effects on the pulmonary vasculature and RV remodeling. Post-menopausal women may lose this protection, increasing susceptibility to PAH and RHF.

Women, particularly younger ones, are more likely to develop idiopathic PAH, a major cause of isolated RHF. Female-dominant connective tissue diseases such as systemic sclerosis or lupus also increase RHF risk.

Common causes of RHF in women are:

1. Left heart failure (LHF) and especially heart failure with preserved ejection fraction (HFpEF) are more common in women.
2. Pulmonary hypertension (idiopathic, connective tissue disease-associated)
3. Valvular disease (TR, often secondary)
4. Congenital heart disease
5. Peripartum cardiomyopathy
6. Obesity and sleep apnea (more prevalent in older women, linked to PH and RHF)

**Fig. 1:**
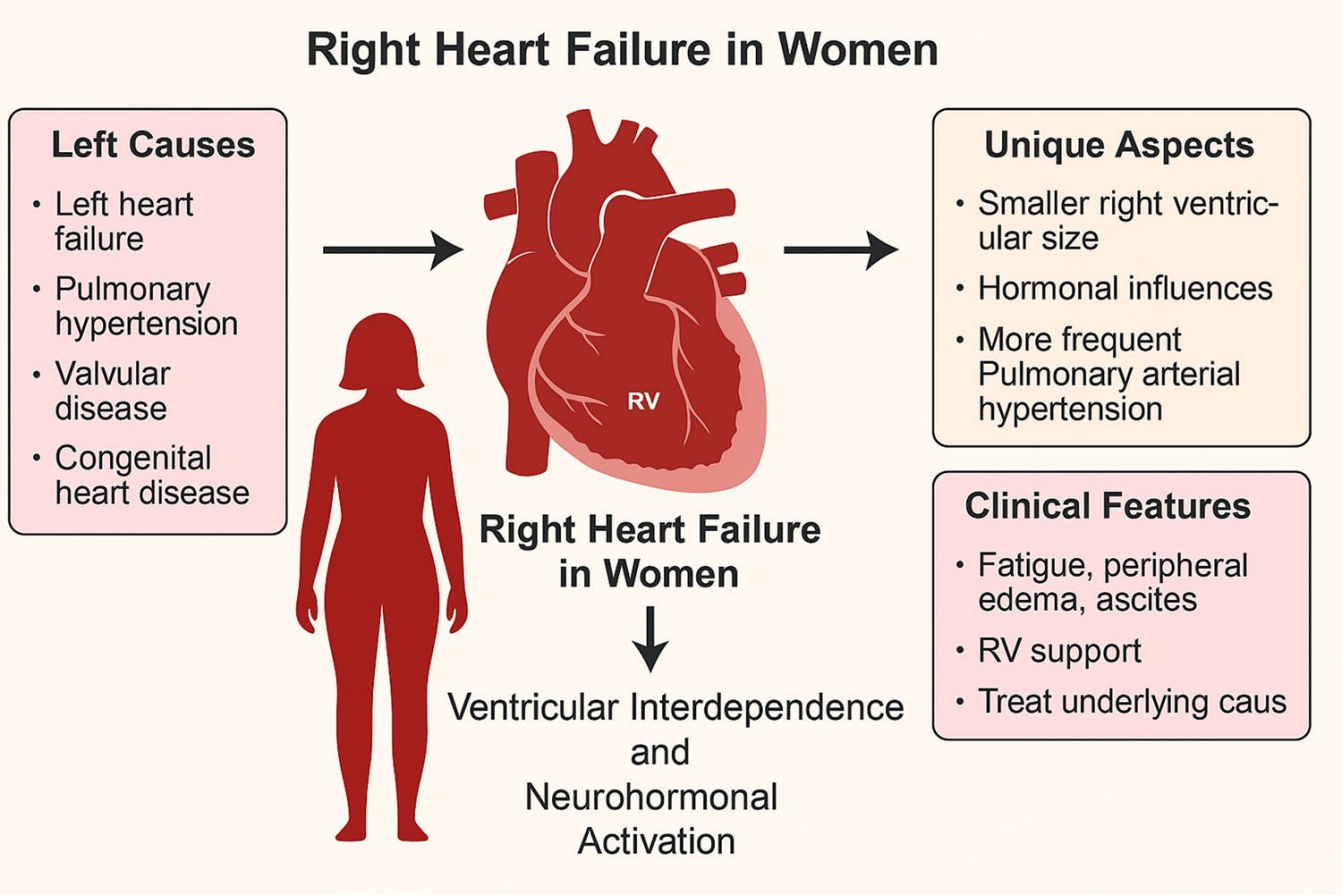
Right heart failure in women. The degree of right heart failure may influence outcomes after tricuspid interventions. Generated by AI

All these factors play a role in outcomes after tricuspid interventions. The clinical features are fatigue, peripheral edema, ascites, hepatomegaly, and jugular venous distension. These may be under-recognized in women because symptoms like fatigue and abdominal bloating are often attributed to non-cardiac causes (Fig. 1).

### Heart failure hospitalization and functional status

The TriValve registry assessed 556 patients undergoing TTVI and found comparable outcomes between sexes regarding 1-year freedom from all-cause mortality (80.9% in women vs. 77.9% in men), and in HF hospitalization, NYHA class III–IV (*p* = 0.17) and TR severity > 2 + at last follow-up. Multivariable Cox regression weighted by IPTW showed improved 1-year survival after TTVI compared to OMT in both women and men [31].

### Transcatheter tricuspid valve implantations

The only CE-approved tricuspid catheter valve is the Evoque™ (Edwards Lifesciences, CA). Screening for transcatheter tricuspid valve implantations involves evaluating patients anatomically and clinically.

A report for valve implantation including 149 patients had a failure rate of 74%, female inclusion of 54%, and median age 79 years. However, the patients who underwent TTVI had significant 30-day functional improvements, which were superior to the improvement in those who screen failed for TTVI and subsequently underwent “bailout” T-TEER. The numbers of females were 26/38 TMVI, 11/26 T-TEER, so the percentage of females treated with the device was 37/64 = 58% [32].

## Conclusions

Women are under-represented in clinical trials, also in trials for transcatheter mitral and tricuspid interventions. In many trials, there is no specification for the outcomes for the different sexes. With the fact that some trials have major male representation, the mitral and tricuspid trials have, on average, a 50/50% sex distribution. Clinical trials are used for establishing guidelines, and guidelines are not sex specific. For the future, we should focus more on the differences between men and women to better establish guidelines and customize treatment options.

## Data Availability

NA.
